# Computational prediction of replication origins: a comparative review of methods, benchmarks, and trends from heuristics to deep learning

**DOI:** 10.1093/bib/bbag315

**Published:** 2026-06-19

**Authors:** Haidar Jomaa, Rida Assaf

**Affiliations:** Computer Science, American University of Beirut, Bliss, 1107 2020, Beirut, Lebanon; Computer Science, American University of Beirut, Bliss, 1107 2020, Beirut, Lebanon

**Keywords:** Ori prediction, DNA replication, machine learning, deep learning

## Abstract

Origins of DNA replication (*Ori*) are genomic loci where DNA synthesis begins, essential for bacterial viability, eukaryotic development, and genome integrity. While early computational methods relied on compositional skews and motif heuristics, recent years have seen a shift toward artificial intelligence approaches. This review systematically surveys *Ori* prediction methods published through August 2025, integrating biological background with curated datasets, benchmarks, and a classification of computational strategies from rule-based models to deep learning. We summarize performance across organisms, cell types, and platforms, and highlight key trends, challenges, and opportunities in benchmarking, interpretability, and cross-species generalization—providing both an accessible entry point for practitioners and a roadmap for future methodological development.

## Introduction

DNA replication is an indispensable cellular process in which genomes are duplicated prior to division. Accurate initiation of this process is essential for faithful genome duplication and long-term genomic stability across all domains of life. It begins at specific genomic loci called origins of replication (Ori), where initiator proteins assemble and establish bidirectional forks [1, 2]. Correct identification of these loci is a prerequisite for understanding how replication is regulated, coordinated with the cell cycle, and adapted to different developmental and physiological contexts. Despite notable domain-specific differences among bacteria, archaea, and eukaryotes, origin usage follows a conserved logic: initiator recognition of DNA, helicase loading, and replisome assembly proceed in a regulated temporal program [3]. Small genomes (such as bacterial chromosomes, plasmids, and viral DNA) typically rely on a single origin to ensure complete replication each cycle [4]; by contrast, large eukaryotic genomes deploy many origins per chromosome to finish duplication within S phase [5, 6]. This architectural diversity makes experimental mapping of origins challenging and shows the importance of accurate genome-wide Ori prediction approaches. Tight spatial and temporal control of origin firing safeguards genome stability; its deregulation induces replication stress, DNA damage accumulation, and disease, including tumors, highlighting the need to accurately characterize and predict replication origins [7, 8]. Therefore, reliable identification of replication origins is a foundational step toward elucidating replication mechanisms and enabling large-scale genomic analyses. In mammals, replication initiates from tens of thousands of sites per cell cycle (estimated $\sim$30 000–50 000 in humans), activated in a regulated temporal program across S phase [9, 10]. However, unlike sequence-defined origins in some yeasts, human origins are not specified by a single strict consensus motif; instead, origin usage is shaped by chromatin context, DNA features, and origin firing factors, and it remains unclear why only a subset of potential sites fire in a given cell type or condition [11, 12].

Origin architecture and initiation mechanisms diverge across life. Among eukaryotes, origin specification ranges from relatively sequence-encoded origins in budding yeast to more flexible, redundant origins in metazoans. In *Saccharomyces cerevisiae*, many origins coincide with autonomously replicating sequences (ARS) whose activity depends on defined sequence elements, enabling early genome-wide origin catalogs [13, 14]. In contrast, in humans and most multicellular eukaryotes, origin positions are more cell-type dependent and strongly modulated by chromatin environment rather than a conserved DNA consensus [11, 12]. Bacterial *oriC* regions often contain conserved DnaA boxes flanking an AT-rich unwinding element [15]. Archaeal origins feature origin recognition boxes (ORBs) bound by Orc1/Cdc6 family initiators, as previously characterized [16].

Experimentally pinpointing origins remains labor-intensive and system-specific. The definition of a replication origin is itself assay-dependent. Early low-throughput methods, such as autonomously replicating sequence (ARS) assays, 2D gel electrophoresis, and plasmid-based replication tests, identified discrete initiation sites under specific experimental conditions [13, 17]. Modern high-throughput approaches—including short nascent strand sequencing (SNS-seq), Okazaki fragment sequencing (OK-seq), chromatin immunoprecipitation sequencing (ChIP-seq), and Repli-seq capture complementary but non-identical aspects of replication initiation, ranging from origin licensing and firing directionality to replication timing [18–20]. As a result, different assays can yield partially overlapping, and sometimes divergent, origin catalogs [11, 12]. Common approaches such as chromatin immunoprecipitation (ChIP) of initiation factors, ChIP-seq, and surface plasmon resonance (SPR)-based assays-require organism-tailored protocols and substantial resources [21, 22]. Consequently, *in silico* prediction has become a practical complement: it accelerates genome annotation, prioritizes candidates for validation, and enables comparative analyses across taxa.

Historically, computational origin discovery relied on compositional asymmetries (Guanine-Cytosine/Adenine-Thymine (GC/AT) skew, Z-curve) and motif heuristics (e.g. initiator binding boxes) [23, 24]. These strategies work well in many bacteria but struggle in archaeal and eukaryotic genomes that use multiple, context-dependent origins lacking simple sequence signatures. Over the past decade, artificial intelligence (AI) has reshaped the landscape: engineered sequence/structure features paired with supervised classifiers gave way to embedding-based encoders and deep neural models that ingest sequence data and learn hierarchical representations, often reducing reliance on hand-engineered features [25, 26]. Yet many earlier predictors offered limited cross-species accuracy or usability (often lacking robust, maintained web servers), and recent progress from 2021 to 2024 is scattered across datasets with heterogeneous construction and reporting.

While replication origin prediction has been reviewed previously [25–30], these works have either focused on specific taxa, heuristic programs, or early machine learning (ML) pipelines, and none capture the more recent deep learning (DL) developments or provide a unified view across both prokaryotes and eukaryotes.

We address this gap by consolidating the major datasets and databases, mapping model pipelines and results, and assembling comparative performance tables across species. Accordingly, this review focuses on the rapid evolution of artificial intelligence-based approaches for replication origin prediction from the early 2010s to 2025, while providing historical context from the heuristic era of the early 2000s. Finally, we propose an updated framework for future ORI prediction, building on the benchmark concept first introduced by Chou [31] for ML development. This framework emphasizes clear benchmarking, principled feature design, transparent pipelines, and interpretability, with the goal of enabling predictors that are both accurate and broadly transferable.

## Datasets, databases, and benchmarks

### Curated databases

As computational capabilities advanced, additional biological databases emerged, including those focused on replication origins [32, 33]. We present to you a list of those resources related to ORI in Supplementary Table S1.

### Benchmark datasets

ML models require positive origin sequences and negative (non-origin) samples. For a more detailed description refer to Supplementary Table S2. Popular benchmark datasets include:

- **O1 benchmark [23]**: Based on OriDB [34], this dataset was constructed as the first benchmark dataset of ORI.
- **O2 benchmark [35]**: Following that, O2 was constructed as the second yeast benchmark dataset. Most early predictors (e.g. iORI-PseKNC, iROS-gPseKNC) [35, 36] used this dataset of 300-bp sequences derived from *S. cerevisiae*. Positive sequences come from confirmed OriDB entries; negatives are randomly extracted from upstream regions before removing high-homology sequences using CD-HIT [37].
- **Human dataset—O3 benchmark [38]**: The iOri-Human predictor assembled confirmed human origin sequences (300 bp) from DeOri’s HeLa dataset and extracted negative sequences upstream and downstream of origins. Redundant sequences were removed using CD-HIT.
- **O4 benchmark [39]**: Another dataset was constructed from *S. cerevisiae*, where the focus was on creating negative samples.
- **O5 benchmark [40]**: The second most popular benchmark, constructed as a combination of four different yeast species from DeOri [41], including *Saccharomyces cerevisiae, Schizosaccharomyces pombe, Kluyveromyces lactis, Pichia pastoris*.
- **Cross-species eukaryotic dataset [42]**: To train generalized models, iORI-Euk compiled a large number of origins from DeOri and an equal number of negative sequences. Each origin was truncated to 300 bp and negative samples were taken from upstream and downstream flanking regions, then used CD-HIT to remove redundant samples.
- **Epigenomic information dataset [43]**: K562 ORIs were taken as positives from GSE28911 (BED; nascent-strand sequencing). Guided by the observed distributions, 95% of ORIs are 100–800 bp long and over 60% of adjacent ORIs lie <10 kb apart—they defined negatives as 800–1000 bp inter-ORI fragments located only between ORIs separated by >10 kb. Final validation used the held-out set and an external K562 ORI collection from Replication Domain [44].
- ***Saccharomyces cerevisiae* ARS dataset [45]**: A yeast-specific benchmark by retrieving the S288C (R64-2-1) reference genome and annotations [ARSs, ARS consensus sequences (ACSs), andintergenic] from SGD, aggregating ARS records from OriDB and DeOri, and incorporating experimentally supported sets from 2D-gel assays, plasmid-based assays, and miniARS-seq. Each origin was standardized to 300 bp; negatives were length-matched intergenic fragments excluding ARSs/ACSs. Redundancy was reduced with CD-HIT.
- **Ori-Deep benchmark dataset [46]**: Built from DeOri’s most recent, well-annotated origins, this benchmark follows iORI-Euk/Stack-ORI [42, 47] protocols and excludes yeast. Positive samples are experimentally validated ORIs, each truncated to 300 bp. Negatives are length-matched flanking fragments drawn upstream (−600 to −300 bp) or downstream (+300 to +600 bp) of each origin. Duplicate sequences were removed to ensure uniqueness.
- **iORI-ENST benchmark dataset [48]**: Augmenting the O2 dataset, this benchmark adds two datasets from *Arabidopsis thaliana*. Although this benchmark combines different benchmarks from many areas, you can access it from this paper directly, hence its placement.

Importantly, the experimental assay used to define replication origins directly shapes the resulting training labels and, consequently, model generalization. Different high-throughput techniques capture distinct aspects of replication initiation: nascent-strand–based assays emphasize actively firing origins, replication-timing approaches delineate broader replication domains, and ChIP-seq–based methods reflect protein binding or chromatin accessibility. As a result, benchmark datasets may encode assay-specific biases, and models trained on a single modality risk learning protocol-dependent signals instead of broadly conserved origin determinants.

## Methods landscape

**Figure 1.**
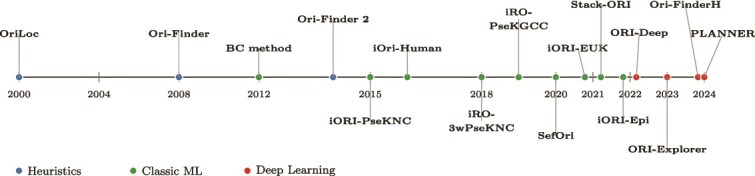
Timeline of major ORI prediction methods, showing the shift from heuristic approaches to ML and DL models.

**Figure 2.**
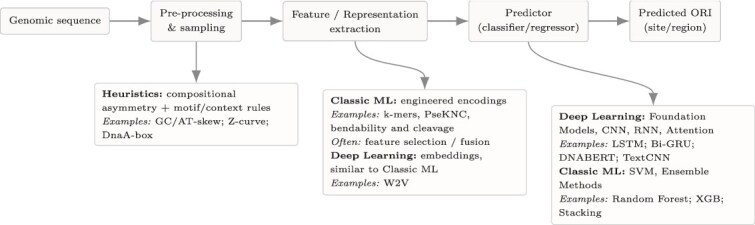
Conceptual overview of ORI prediction methodologies, illustrating the transition from compositional and motif-based heuristics to classical machine learning with engineered features and recent deep learning/foundation-model approaches based on learned sequence representations.

A high-level timeline of origin prediction methods is shown in Fig. 1, tracing the field’s progression from compositional heuristics to classical ML and recent DL approaches. In the sections that follow, we expand on each category in detail. While Fig. 1 summarizes the chronological development of individual predictors, Fig. 2 provides a high-level abstraction of the shared pipeline and the evolving design principles underlying these methods, ranging from heuristic signal detection to feature-engineered ML and modern representation-learning approaches.

### Classical/compositional approaches

Early computational approaches to predict bacterial origins of replication (*oriC*) leveraged compositional asymmetries in DNA sequences. One of the first methods was GC skew analysis, which, along with AT skew, typically exhibits a characteristic V-shaped inversion near the replication origin and terminus. These polarity shifts arise from strand-specific mutational pressures during replication and were first observed in bacterial chromosomes by Lobry [49, 50], and the computer program *Oriloc* was then developed by Frank and Lobry [51]. However, such skew-based methods often only approximate the origin and may mispredict it [52].

Precise oriC mapping needs more than GC-skew: verify the skew switch by locating a species-specific cluster of DnaA-box motifs and the nearby dnaA gene-after first defining the organism’s DnaA-box consensus. A complementary GC-content method, the GC profile, summarizes cumulative GC variation across a candidate origin [53]. Still, these methods faced several limitations: they typically pinpointed a central position rather than delineating the true boundaries of the *oriC* region, and they lacked species-specific motif detection.

To address these limitations, Ori-Finder was built by integrating multiple features: Z-curve disparity, species-aware DnaA-box clustering, localization of the dnaA gene, phylogenetic context, and indicator genes frequently adjacent to bacterial *oriC* [24]. It accepts annotated or unannotated genomes, lets users select or define species-specific DnaA-box motifs with mismatch tolerance, ranks intergenic regions, and reports explicit *oriC* boundaries with integrated plots and tables. The predictions are verified against a few experimentally validated bacterial origins [15].

For archaeal genomes, Ori-Finder 2 extends the framework with ORB motif discovery and scanning: taxon-specific ORB PWMs are derived from DoriC using MEME, then applied with FIMO; REPuter identifies repeats/palindromes; and ZCURVE/Glimmer plus local BLAST support unannotated inputs [54]. The tool can report multiple origins (e.g. in haloarchaea), but may not recover all of them; in a benchmark of 13 archaeal chromosomes, sensitivity and precision were $\sim$66.7% and $\sim$62.1%, respectively.

For bacterial plasmids, OriV-Finder [55] implements an RIP-centric pipeline: homology detection is done by MMseqs2/HMMER3; intergenic regions are then scored for oriV features (BLAST hits to curated oriV regions, ColE1-like modules via Infernal, iterons by k-mer/entropy, AT-rich segments via a Z-curve variant, and conserved motifs), with candidates prioritized (Types 1–3) by replication initiation protein (RIP) proximity and evidence. It addresses the paucity of plasmid oriV annotations in DoriC and, on a 327-plasmid benchmark (380 annotated oriVs), recovers 366 oriVs (Type 1) versus 81 by *Ori-Finder* 2022 [56].

Beyond bacteria and archaea, early eukaryotic work focused on budding yeast. In *S. cerevisiae*, a nucleotide–correlation measure more precisely delineates replication origins than GC skew. In budding yeast, Oriscan examines 268 bp windows containing the T-rich ACS plus an adjacent $\sim$30 bp A-rich tract, then ranks candidates by predicted activity-recovering a large fraction of genomic origins with near-perfect specificity [57]. While effective, Oriscan produced many candidates due to the degeneracy and genomic abundance of ACS-like motifs and did not by itself define precise origin boundaries.

Combining both prokaryotic and eukaryotic under one signal-processing scheme, iCorr [58] encodes all four nucleotides and computes an autocorrelation-based signal to call replication origins objectively—using peaks for bacteria and zero-crossings for *S. cerevisiae*—thereby avoiding manual plot inspection. Unlike earlier composition/signal approaches (GC-skew, Z-curve, GCC, AT-excursion, gCorr, etc), iCorr leverages all four bases rather than a single-base encoding, yielding sharper and more window-robust localization in prokaryotes. Evaluated on 38 bacterial genomes [59] and the 16 yeast chromosomes (OriDB) [34], it outperforms gCorr on bacteria but attains only modest, low-precision results in yeast-reflecting the challenge of eukaryotic origin discovery.

Despite these advancements, non-learning-based methods remain limited in their applicability to complex or rearranged genomes-often yielding false positives or negatives, particularly in eukaryotes where origin-defining sequence features are sparse and chromatin context dominates.

### From heuristics to learning: why machine learning emerged

While compositional asymmetries such as GC skew and motif-based heuristics enabled early *in silico* prediction of origin sites, these approaches were limited in scope. They often failed to account for genomic context, suffered from poor resolution in complex genomes, and lacked adaptability across species. As genome annotations and high-throughput origin mapping datasets became increasingly available in the 2010s, supervised ML methods emerged as a natural extension. By leveraging statistical regularities within known origin sequences, beyond hand-specified motifs, these models promised improved accuracy, adaptability, and scalability. In the following section, we chronicle the progression of ML-based origin predictors, emphasizing how each iteration addressed prior limitations, expanded feature representation, or improved model performance. Supplementary Table S2 summarizes the benchmark datasets that have enabled comparative evaluation of these ML-based methods.

### Traditional machine learning with engineered features

To address the limitations of sequence-only approaches for predicting origins of replication in *S. cerevisiae*, two DNA structural properties (bendability and hydroxyl radical cleavage intensity) were investigated as discriminative features [23]. They observed that both measures were significantly lower in core replication regions compared with flanking linker regions. Building on this insight, the authors created a benchmark dataset from the OriDB database, encoding each sample into a 10D vector via a 50 bp sliding window for structural feature extraction. A support vector machine (SVM) was trained and evaluated using jackknife cross-validation.

Building on prior work, iORI-PseKNC was introduced, which integrates local and global sequence-order information via pseudo k-tuple nucleotide composition (PseKNC) [35]. Each sequence was encoded with PseKNC, which captures both short-range $k$-mer composition and long-range correlations in six local DNA structural parameters. These features were fed into an SVM, evaluated by rigorous jackknife cross-validation. When benchmarked against the bendability+cleavage intensity (BC) method under identical conditions, iORI-PseKNC demonstrated superior auROC, demonstrating the benefit of incorporating long-range structural correlations.

Expanding upon the PseKNC framework, iROS-gPseKNC was proposed, introducing dinucleotide position-specific propensity (DPSP) to enhance ORI recognition [36]. They constructed a $16 \times 299$ DPSP matrix capturing frequency differences of each dinucleotide at every position between positive and negative sets. The resulting vectors were combined with PseKNC features and fed into a random forest (RF) classifier. Jackknife cross-validation showed marked improvements over BC and iORI-PseKNC.

As the first ORI predictor tailored for human DNA, iOri-Human was introduced [38], employing an enhanced type-1 PseKNC representation to capture both $k$-mer composition and long-range sequence-order effects. Multiple classifiers were tested under jackknife cross-validation, with RF outperforming SVM, naïve Bayes, KNN, and decision trees.

Focusing on yeast ORI prediction, a predictor was developed to address the limitation of prior methods [40]. The method extends PseKNC to a three-window formulation, incorporating GC asymmetry into the feature space, which is then processed into composite vectors for an RF classifier.

With iORI-PseKNC2.0, a new approach was proposed to address the low accuracy of iORI-PseKNC and the slow runtime of iROS-gPseKNC, targeting ORI prediction in *S. cerevisiae* [60]. They replaced the traditional type-1 PseKNC with a type-2 formulation, yielding a 90D feature set. A two-stage feature selection pipeline was applied before training an SVM classifier to avoid high-dimensionality curse and improve the accuracy.

Building on iRO-3wPseKNC, iRO-PseKGCC was introduced, embedding GC skew information directly into the PseKNC framework for improved ORI prediction [61]. They formulated PseKGCC to capture strand asymmetry more explicitly. These features were processed by an ensemble of fused RFs, combining outputs from multiple RF with jackknife cross-validation showed clear gains over iRO-3wPseKNC.

Targeting eukaryotic ORI prediction, particularly in yeast, SefOri was proposed [62]. They extracted sequence-order information via PseKNC and systematically explored six feature selection methods alongside seven classifiers. Their final pipeline applied SVM-RFE [63] to refine the PseKNC-derived features, followed by a BPNN for classification [64]. Evaluated with five-fold cross-validation, SefOri outperformed previous studies, confirming that optimal feature subset selection can significantly boost prediction accuracy.

For eukaryotic ORI prediction, an Xtreme Gradient Boosting (XGBoost) framework was introduced that integrates state-of-the-art sequence features with NLP-inspired embeddings [65, 66]. They extracted traditional sequence-order descriptors via PseKNC and semantic representations via FastText’s continuous bag-of-words model. These complementary feature sets were concatenated and input to an XGBoost classifier.

Marking a major step toward broad eukaryotic ORI prediction, iORI-EUK was introduced, expanding coverage beyond yeast and human genomes [42]. Leveraging DeOri, they built a multi-species benchmark dataset, partitioned into training and held-out test sets, with five-fold cross-validation guiding model selection. Three encoding schemes were explored: $k$-mer composition (340D) capturing oligonucleotide frequency patterns, binary encoding (1200D) mapping nucleotide positions to discrete indicators, and a fused representation (1540D). An SVM classifier trained on the $k$-mer features achieved the best performance, surpassing the best previous studies for the respective species on the independent test set, and establishing a new standard for cross-species eukaryotic ORI identification.

For prokaryotic replication origins, gammaBOriS [67] introduced a motif-based SVM framework tailored to Gammaproteobacteria. Addressing the limitations of nucleotide-disparity methods, the pipeline employed LS-GKM and gkm-SVM [68, 69] to classify $k$-mer patterns: candidate fragments centered on seed motifs were scored, thresholded to handle class imbalance, and used to build the BOriS database by scanning RefSeq and UBA genomes [70, 71].

To improve eukaryotic ORI prediction, especially in yeast, yORIpred [72] combined eight encoding schemes with diverse classifiers using a two-step feature selection and iterative representation learning pipeline to assess cross-species transferability. Building on broader accuracy concerns, iORI-ENST [48] employed mono-nucleotide binary encoding and dinucleotide spatial autocorrelation for sequence representation, applied Elastic Net for feature selection, and used a stacking strategy to integrate base classifiers into a meta-model.

For cell-specific ORI prediction, Stack-ORI [47] engineered 12 feature encodings, refined them through two-step selection, and integrated the most informative features via a stacking ensemble of XGBoost base learners, with SHAP analysis highlighting the top contributors. Advancing eukaryotic ORI prediction in *S. cerevisiae*, Ori-Finder 3 [45] combined ACS motif and AT-rich scanning with Z-curve mapping sequences into 3D curves reflecting nucleotide distribution asymmetries, screened candidates through ARSbench, and applied SVM classification, addressing prior issues of length and boundary sensitivity.

Emphasizing the role of epigenomic context, iORI-Epi [43] integrated transcription factor motifs, histone modifications, and chromatin spatial interactions into ORI prediction. ORIs were annotated using ChIP-Seq data [21] from ENCODE [73], combined with DNA motifs and chromatin interaction features to yield 626 descriptors, which were reduced to 60 via recursive feature elimination [74] and classified with an RF.

ORCA was introduced to address limitations of existing tools such as OriLoc and Ori-Finder [75]. Building on Ori-Finder’s use of Z-curve, GC-skew, and dnaA-box localization, ORCA incorporates an RF classifier to assign confidence scores to candidate origins. Trained on data from DoriC and evaluated on two independent test sets, the model consistently matched or surpassed Ori-Finder while enabling genome-wide and batch oriC detection.

Despite considerable progress, traditional ML methods remained limited by their dependence on manually designed features, which are often error-prone and reliant on domain expertise; to resolve these issues many use feature selection processes which add more complexity to the pipeline.

### Synthesis: what did traditional machine learning achieve?

Collectively, traditional ML methods introduced several innovations to origin prediction. They formalized the encoding of DNA sequences via $k$-mer statistics, structural descriptors, and signal transformations; established organism-specific benchmarks and reproducible evaluation pipelines; and enabled ensemble modeling and meta-learning for improved accuracy. Yet, these models remained tethered to manually engineered features that required biological insight, often entailed iterative tuning, and struggled to generalize across species or datasets. As benchmarks expanded in scale and diversity, the limitations of these handcrafted pipelines became more evident, paving the way for end-to-end representation learning with deep neural networks as shown in Supplementary Table S3.

### Deep learning

The emergence of DL marked a major shift in origin prediction: away from manual feature engineering toward end-to-end learning from raw sequences [76]. Deep neural networks can capture hierarchical structure, long-range dependencies, and positional signals directly from DNA, obviating the need for hand-crafted descriptors. This is especially important in origin prediction, where replication activity is shaped by both local motifs and broader genomic context. In what follows, we review the architectures deployed for ORI prediction-including convolutional, recurrent, and transformer-based models-and describe how embedding layers, attention mechanisms, and multimodal inputs have pushed the field forward.

One of the first DL approaches for eukaryotic ORI prediction was introduced by casting DNA as text [77]. To overcome the limitations of fixed-length inputs and improve accuracy, sequences were split into overlapping 3-mers, embedded via skip-gram word2vec [78], and fed to a width-spanning TextCNN [79]. Building on this, a CNN-with-embeddings pipeline was developed using two 3-mer segmentation schemes [80]: Continuous-TSSS (continuous three-slices sequence segmentation) and Skip-TSSS (skip three-slices sequence segmentation), skip-gram word2vec with negative sampling, and three embedding modes (fixed, trainable, and two-channel), followed by vertical convolutions, pooling, and a softmax classifier for ORI prediction.

Complementary to CNN approaches, ORI-Deep [46] tackles eukaryotic ORI prediction across four species. The authors propose a blended feature–LSTM design: raw sequences are encoded (A=1,C=2,G=3,T=4) and transformed into frequency/position descriptors (PRIM/RPRIM, AAPIV/RAAPIV) plus statistical moments (raw/central/Hahn); these components are concatenated into a single 822-dimensional vector, which feeds an LSTM followed by dense layers [81].

Turning to ORI-Explorer [82], the authors target four eukaryotic species and fuse three hand-crafted encodings (CKSNAP, PCPseDNC, and DCC) with features learned by a deep network (six parallel convolutional layers $\rightarrow$ two Bi-GRUs $\rightarrow$ attention $\rightarrow$ FC) [83, 84] which outputs a prediction of ORI/non-ORI. SHAP [85] is used to rank and select informative features, which are then fed to a CatBoost classifier [86].

Ori-FinderH [87] focuses on human ORIs and couples Z-curve encoding with DL: variable-length sequences are projected into fixed-dimensional Z-space and fed to a stacked architecture (structure blocks, each with self-attention, 1D conv, pooling, then upsampling; followed by a softmax head). Using the iORI-Epi benchmark, it reports strong performance and surpasses iORI-Epi across cell lines, as shown in Supplementary Table S4. Limitations include sequence truncation and epigenetic reliance, and they also introduce a genetic algorithm that uses the model as a fitness function to evolve synthetic 1-kb ORIs from A-only or G-only seeds via point/indel/structural mutations.

Finally, PLANNER was developed, targeting four eukaryotic species and replacing manual feature design with a multi-scale DNABERT ensemble [88, 89]: ORI sequences are tokenized into k-mers (k=3,4,5,6), four k-mer length-specific DNABERTs are fine-tuned, and a cell-specific ensemble (soft voting or a “blending” scheme) is selected before evaluation. Supplementary Table S5 presents how using the same DeOri benchmark as iORI-EUK, they report higher MCC and AUC than prior work.

### Synthesis: trends and tensions in deep learning for ORI prediction

DL has substantially improved origin prediction performance across several benchmarks, particularly in complex eukaryotic genomes. Embedding-based models, especially those using skip-gram or DNABERT encodings, have outperformed traditional pipelines by capturing distributional and contextual properties of sequences without relying on predefined motifs or structural priors. Attention mechanisms, such as those used in ORI-Explorer and PLANNER, have begun to bridge the gap between accuracy and interpretability by highlighting informative subsequences.

Yet challenges persist. Deep models often require large, balanced, and well-annotated datasets, which remain scarce for many non-model organisms. Overfitting to species or assay-specific features is a risk, and generalization across cell types or experimental platforms is not yet reliably achieved. This risk is amplified when training labels are derived from a single experimental modality, as deep models may internalize protocol-specific biases instead of biologically conserved initiation mechanisms. Furthermore, while interpretability tools like SHAP and attention maps offer insight, these techniques are not yet standardized, and biological validation remains limited. In short, while DL has delivered technical advances, its integration with biological insight and experimental design remains an open frontier.

### Interpretability and biological validation in deep ORI models

Interpretability remains essential given the biological complexity underlying origin determinants. Classical models based on GC skew or motif searches are naturally interpretable, offering explicit hypotheses regarding DnaA boxes or ORB motifs [24, 49, 50]. Feature-engineered approaches, such as k-mer frequencies or physicochemical descriptors, facilitate ranking of informative sequence patterns; for instance, iORI-Epi demonstrated that epigenomic marks like H3K4me1, H4K20me1, and ETS1 protein status serve as strong predictors [43]. ML frameworks often facilitate interpretability: tree-based classifiers provide transparent decision rules, while SVMs yield feature weights that can be directly assessed [35, 47, 87].

DL methods, by contrast, pose greater challenges. To address this, ORI-Explorer employed SHAP values [85] to disentangle the contributions of deep modules from handcrafted descriptors [82]. Attention mechanisms in transformer and self-attention architectures [84] further enhance interpretability by highlighting influential subsequences, thereby enabling mapping back to candidate motifs and capturing long-range dependencies [80, 88]. However, these interpretability tools come with important caveats. SHAP explanations depend on background distribution choices and feature independence assumptions, and attribution scores may be unstable or misleading when features are highly correlated. Similarly, while attention weights offer intuitive visualizations, they do not necessarily constitute faithful explanations of model decisions; multiple studies have shown that attention scores can be redistributed without materially affecting predictions, limiting their causal interpretability [90, 91]. As such, explainability analyses should be interpreted as heuristic insights rather than definitive explanations.

Nonetheless, deep models can inadvertently learn species-specific biases or reflect experimental artifacts, underscoring the necessity of biological validation through wet-lab experimentation. For deep models in particular, attribution analyses should be complemented by orthogonal experimental evidence, such as overlap with independently mapped origins or consistency across assay modalities, to avoid over-interpreting model-internal signals. As such, interpretability is not only desirable but imperative for trustworthy ORI prediction. Moving forward, future studies are strongly encouraged to incorporate dedicated sections on model explainability, ensuring that computational predictions remain biologically meaningful and actionable.

## Comparative performance of origin predictors

The growing diversity of origin prediction models has made performance comparison increasingly important, and increasingly complex. Supplementary Tables S4–S6 present a consolidated summary of predictive performance across multiple benchmarks, spanning prokaryotic, yeast, plant, and mammalian systems.

Direct comparison of predictor performance remains challenging due to heterogeneous datasets, inconsistent negative sampling strategies, and varying evaluation protocols. Generalization across species remains limited. Most models are trained and evaluated on organism-specific datasets, with few cross-genome validation studies. Moreover, methodological inconsistencies make it difficult to compare reported performance directly. These include variation in negative set construction (e.g. randomly sampled non-origin regions versus biologically matched controls), differences in sequence window sizes, and inconsistent use of cross-validation versus held-out test sets. Even the choice of metrics-AUC, MCC, or accuracy-varies between studies, and not all models report the same metrics. So while the comparative table offers useful trends, its values should be interpreted as indicative rather than definitive. Nonetheless, these results reveal several key trends and limitations.

These comparisons also mask important trade-offs between *datasets* and *model families*. Dataset design strongly conditions apparent generalization: models trained on multi-species collections (e.g. iORI-EUK) or evaluated with cross-species transfers are more informative about robustness than single-organism splits [82, 88], while negative-set choices can inadvertently simplify the task (random non-origin windows) or better stress biological specificity. Methodologically, heuristic and motif-based tools are typically fast and transparent but limited when origins lack stable sequence signatures; classical ML improves flexibility while retaining partial interpretability via engineered features and feature-importance analysis; DL and foundation-model encoders can learn higher-order context and transfer better with scale, but at the cost of higher computational demands and reduced intrinsic interpretability. Notably, most studies do not report training hardware or runtime, making compute–accuracy trade-offs difficult to quantify and reinforcing the need for standardized reporting in benchmarking.

First, variation across models on the same benchmark remains high. Supplementary Table S6 reveals that on the O2 dataset derived from *Arabidopsis*, reported accuracies range from 68.6% (Ori-Finder 3) to 98% (iROS-gPseKNC), with corresponding differences in MCC. On yeast benchmarks such as O5, newer embedding-based models (e.g. SefOri and Word Embeddings) reach near-ceiling MCC values above 0.93, while older models like iRO-3wPseKNC lag behind.

Second, modern DL models-particularly ORI-Deep, ORI-Explorer, and PLANNER-consistently outperform traditional feature-based methods on complex multicellular eukaryotic datasets, particularly those in the iORI-EUK collection. This trend is clearly illustrated in Supplementary Table S5, particularly for human (K562 and MCF7) and mouse (ES, MEF, and P19) cell types, where PLANNER typically achieves the highest AUC and MCC values. For instance, in mouse embryonic stem cells, PLANNER reaches an MCC of 0.84 and AUC of 0.976, outperforming other approaches. Similar patterns hold across *Drosophila* and *Arabidopsis*, suggesting robustness across species and contexts. Architecturally, the strongest performers tend to combine (i) representations that capture both local sequence patterns and longer-range dependencies, and (ii) training setups that better support transfer across species or cell types. For example, hybrid deep networks that integrate convolutional blocks with recurrent/attention mechanisms can jointly model short motifs and broader contextual signals [82], while transformer-based encoders benefit from large-scale pretraining and attention-driven context aggregation, which often improves robustness [88]. These design choices help explain why the leading deep models dominate on complex multicellular benchmarks.

Third, stacking and ensemble methods show consistent, though not always superior, improvements over their constituent models. Their performance tends to be close to the best model in each group but not necessarily better. This is apparent in Supplementary Table S5, where Stack-ORI performs close to top-scoring models but does not uniformly surpass them. Meanwhile, embedding-based methods appear to gain ground primarily in lower-resource or cross-species settings such as *P. pastoris* and *K. lactis*.

Fourth, models trained with epigenomic data (iORI-Epi and Ori-FinderH) demonstrate the potential of incorporating chromatin context. On shared benchmarks, Ori-FinderH outperforms iORI-Epi across all human cell lines tested, achieving AUC values as high as 0.987 in HCT116. These results support the growing view that replication origin activity in higher eukaryotes is influenced by more than sequence alone.

In summary, predictive performance has clearly improved from heuristic and motif-based tools to deep neural models with context-aware inputs. The best-performing models now reach near-ceiling accuracy on several curated benchmarks. Yet the field still lacks harmonized evaluation protocols, standardized metrics, and a shared understanding of biological relevance and generalizability. Future progress will require not only better models, but also better benchmarks and a shift toward testing how these tools perform across species, conditions, and validation settings.

## Open challenges and future directions

**Figure 3.**
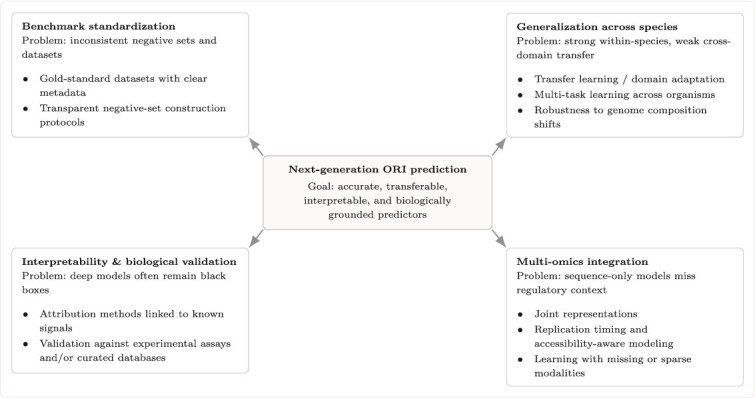
Conceptual roadmap of open challenges and future directions in *in silico* ORI prediction. Key priorities include benchmark standardization, cross-species generalization, interpretability and biological validation, and integration of multi-omics signals, alongside emerging trends such as foundation models and data-centric evaluation.

The history of *in silico* origin prediction reflects a broader trajectory in computational biology: from handcrafted signals and statistical rules to learned representations and AI-powered models. Each stage in this progression has brought methodological innovation, new applications, and fresh challenges, as summarized by Fig. 3.

Despite rapid progress, several open challenges remain unresolved in computational origin prediction. First, the field lacks universally accepted benchmark datasets. The construction of negative sets in particular varies significantly across studies. Establishing gold-standard datasets with clear metadata on experimental methods, sequence quality, and species diversity would substantially improve model comparison and reproducibility.

Second, generalization across species remains weak. Most high-performing models are evaluated within species-specific contexts; few attempt cross-species or cross-cell-type prediction. Transfer learning, domain adaptation, and multi-task training offer promising directions but remain underexplored.

Third, interpretability is uneven across methods. Classical approaches and tree-based models offer some insight into decision-making, but most DL methods remain black boxes. For a detailed discussion of interpretability methods and their biological relevance, see Supplementary Text  *Interpretability and Biological Validation*.

Finally, integration with multi-omics, such as chromatin accessibility, replication timing, or 3D genome conformation, remains in its infancy. Most predictors operate solely on DNA sequence, limiting their ability to capture regulatory nuance. Future models should explore joint representations that span sequence and epigenomic space, ideally grounded in experimentally validated data. This will be crucial not only for understanding replication origins but also for their application in synthetic biology, evolutionary genomics, and disease modeling.

## Conclusion

We began this review with sequence asymmetries, moved to motif-based tools, transitioned through engineered ML classifiers, and finally to DL and transformer-based predictors. Each wave addressed key challenges but also introduced new ones.

Alongside methodological innovation, community resources, such as DoriC, OriDB, and DeOri have provided curated repositories of replication origins, while benchmark datasets and evaluation frameworks have enabled more systematic comparisons across approaches. Our review consolidated these resources, methods, and benchmark results into comparative tables to serve as a practical reference point for future development in the field.

Despite this progress, several critical limitations remain. Benchmarking remains fragmented, with models evaluated under heterogeneous dataset constructions, negative sampling strategies, and validation protocols, which undermines direct cross-study comparison. As a result, the absence of a universally accepted benchmark framework prevents reliable identification of a single “state-of-the-art” predictor and limits objective assessment of generalization performance. Moreover, commonly used benchmark sets are not only limited in size but also require periodic updates as new high-confidence ORIs and profiling assays become available, so that generalization can be assessed under current data realities. At the same time, dataset size and balance remain key challenges: DL models require large training sets, yet available data are unevenly distributed across species and assays-e.g. substantially more work exists for bacteria and yeast than for many other species leading to species-specific biases and limited generalizability, and leaving the field without a clear state-of-the-art consensus.

Moving forward, progress will depend on frameworks that either decisively surpass prior work or open entirely new avenues of investigation. Such frameworks should build on the structure established by existing studies [31], while making the steps explicit: (i) prepare and maintain an up-to-date benchmark dataset, (ii) position the method clearly within prior literature and state concrete improvement goals, (iii) design feature representations and encodings—especially for DL-that minimize hand-crafted assumptions, (iv) specify an end-to-end classification pipeline with principled choices and justifications, (v) report results with rigorous evaluation, interpretability/explainability analyses, and cross-species tests, and (vi) analyse computational cost and scalability to illuminate practical trade-offs. By systematically addressing these aspects, future studies can deliver predictors that are not only more accurate, but also more general and biologically validated. Ultimately, the field will benefit from unified and regularly updated benchmarks, expanded multi-omic data integration, and stronger collaboration between computational and experimental researchers to translate predictive advances into deeper biological understanding.

While earlier reviews addressed bacterial heuristics [27, 28], eukaryotic databases [26], and early ML-based techniques [25], none to date has offered a unified and updated synthesis of both prokaryotic and eukaryotic systems, spanning the full trajectory from pre-AI methods to modern DL. Our work fills this gap and complements existing literature by consolidating benchmarks, methods, and trends across domains.

**Key Points**

- Replication origins (Ori) are critical genomic loci that initiate DNA synthesis and are regulated differently across prokaryotic and eukaryotic systems.
- Early computational approaches relied on compositional biases and motif heuristics; modern methods increasingly leverage machine learning and deep learning.
- This review consolidates benchmark datasets, curated databases, and over a decade of predictive models into a unified comparative framework.
- We highlight key methodological trends, evaluate model performance across species and cell types, and identify persistent challenges in generalization and interpretability.
- Our synthesis provides a roadmap for future development, emphasizing robust benchmarking, cross-species validation, and integration with multi-omics data.

## Supplementary Material

Supplementary_material_bbag315

## Data Availability

This review does not introduce new datasets. All datasets and benchmark resources discussed are either publicly available through the original publications and databases cited (e.g. DoriC, OriDB, DeOri, andiORI-EUK), or were extracted from reported results in the associated papers. Where applicable, we provide reconstructed benchmark tables and evaluation summaries as supplementary material for transparency and reproducibility.

## Use of large language models

Portions of this manuscript were revised and refined with the assistance of a large language model (ChatGPT, OpenAI), including help with restructuring sentences and improving clarity. All scientific content, synthesis, data interpretation, and conclusions were conceived, written, and verified by the authors. No LLM-generated content was used without human oversight. This use complies with the ISCB guidelines for acceptable use of LLMs.
