# Supplementary material for: Computational prediction of replication origins: a comparative review of methods, benchmarks, and trends from heuristics to deep learning
Source: Brief Bioinform. 2026 Jun 19;27(3):bbag315. doi: 10.1093/bib/bbag315 (PMC13280946; doi:10.1093/bib/bbag315)
Supplement: Supplementary_material_bbag315 [file supplementary_material_bbag315.zip › ORI_Review_supplementary_revised_bbag315.pdf]

Supplementary Tables

Table 1. Core curated databases supporting *Ori* prediction across species

Database	Species	Entries (#)	Links	References
OriDB	*S. cerevisiae* and *S. pombe*	829	https://cerevisiae.oridb.org/	[1]
DeOri 10.0	Eukaryotes (animals, plants, fungi, yeast)	9,742,396	http://tubic.tju.edu.cn/deori/	[2]
DoriC 12.0	Prokaryotes (bacteria, archaea) & plasmids	207,555	https://tubic.org/doric/	[3]
BOriS DB	Gammaproteobacteria	25,827	https://boris.mathematik.uni-marburg.de/	[4]

Table 2. Constructed benchmark datasets used for *Ori* prediction

Dataset	ORI (#)	Non-ORI (#)	Source Database(s)	Species
O1 benchmark [5]	322	966	OriDB	*S. cerevisiae*
O2 benchmark [6]	405	406	OriDB	*S. cerevisiae*
O3 (iOri-Human) benchmark [7]	283	282	DeOri	*H. sapiens* (HeLa)
O4 benchmark [8]	251	410	OriDB	*S. cerevisiae*
		502		
		251		
O5 benchmark [9]	340	342	DeOri	*S. cerevisiae*
	338	335		*S. pombe*
	147	147		*K. lactis*
	305	302		*P. pastoris*
iORI-Euk (cross-species) [10]	5,506	5,513	DeOri	*H. sapiens* (HeLa, K562, MCF7)
	7,307	7,307		*M. musculus* (ES, MEF, P19)
	17,335	17,315		*D. melanogaster* (Kc, Bg3, S2)
	1,515	1,515		*A. thaliana*
	268	300		*P. pastoris*
	339	350		*S. pombe*
	136	200		*K. lactis*
iORI-Epi (Epigenomic) [11]	Not Specified	Not Specified	GSE28911, ReplicationDomain	*H. sapiens* (K562)
Ori-Finder 3 (ARS) [12]	380	370	SGD, OriDB, DeOri	*S. cerevisiae*
Ori-Deep benchmark [13]	17,964[1], 13,500[2]	17,949[1], 13,500[2]	DeOri	*H. sapiens, M. musculus, D. melanogaster, A. thaliana*
iORI-ENST benchmark [14]	1,920	1,921	DeOri	*S. cerevisiae, A. thaliana*

[1] Training dataset.
[2] Independent benchmark dataset.

Table 3. Summary of *Ori* predictors

Predictor	Species	Dataset	Algorithm	Encoding Technique
Oriloc	*Bacterial chromosomes*			GC-skew+AT-skew
Ori-Finder	*Bacterial chromosomes*			Z-curve+DnaA-box+dnaA-gene
Ori-Finder 2	*Archaeal genomes*			"Ori-Finder"+ORB motif
OriV-Finder	*Bacterial plasmids*			RIP-centric pipeline
Oriscan	*S. cerevisiae*			Nucleotide-correlation measure
iCorr	*Prokaryotes and eukaryotes*			autocorrelation-bases signal
BC method	*S. cerevisiae*	O1[1]	SVM	Bendability+Cleavage
iORI-PseKNC	*S. cerevisiae*	O2[1]	SVM	PseKNC
iROS-gPseKNC	*S. cerevisiae*	O2	RF	PseKNC
iOri-Human	*H. sapiens*	O3[1]	RF	PseKNC
iRO-3wPseKNC	*S. cerevisiae, S. pombe, K. lactis, P. pastoris*	O5[1]	RF	3-window PseKNC
iORI-PseKNC2.0	*S. cerevisiae*	O2	SVM	PseKNC type-2
iRO-PseKGCC	*S. cerevisiae, S. pombe, K. lactis, P. pastoris*	O5	Ensemble RF	Enhanced PseKNC
SefOri	*S. cerevisiae, S. pombe, K. lactis, P. pastoris*	O5	BPNN	PseKNC+SVM-RFE
XGB	*S. cerevisiae*	O2	XGBoost	PseKNC+Fast-Text
iORI-EUK	*H. sapiens, M. musculus, D. melanogaster, A. thaliana P. pastoris, S. pombe, K. lactis*	iORI-EUK[1]	SVM	k-mer composition
gammaBOriS	*Gammaproteobacteria*	BOriS[1]	LS-GKM	Kmer
yORIpred	*S. cerevisiae, S. pombe, K. lactis, P. pastoris*	O5	SVM	Feature fusion[2]+2-step feature selection+ iterative feature representation
iORI-ENST	*S. cerevisiae, A. thaliana*	iORI-ENST[1]	Stacking	MBE+DSA+Elastic Net
Stack-ORI	*H. sapiens, M. musculus, D. melanogaster, A. thaliana*	iORI-EUK	XGBoost	3 feature categories[3]+feature selection[4]
Ori-Finder 3	*S. cerevisiae*	Ori-Finder 3[1]	SVM	Z-curve
iORI-Epi	*H. sapiens*	iORI-Epi[1]	RF	TF motifs+chromatin interactions
ORCA	*Prokaryotes*	DoriC	RF	Z-curve+GC-skew+DnaA-box
CNN-Based	*S. cerevisiae*	O5	TextCNN	3-mers+W2V
Word Embeddings	*S. cerevisiae, S. pombe, K. lactis, P. pastoris*	O5	CNN	Skip-TSSS/Continuous-TSSS+W2V
ORI-Deep	*H. sapiens, M. musculus, D. melanogaster, A. thaliana*	ORI-Deep[1]	LSTM	Frequency/position descriptors[5]+ statistical moments
ORI-Explorer	*H. sapiens, M. musculus, D. melanogaster, A. thaliana*	iORI-EUK	CNN+Bi-GRU+Attention	CKSNAP+PCPseDNC+DCC+DNN
Ori-FinderH	*H. sapiens*	iORI-Epi	Attention+Genetic Algorithm	Z-curve
PLANNER	*H. sapiens, M. musculus, D. melanogaster, A. thaliana P. pastoris, S. pombe, K. lactis*	iORI-EUK	DNABERT Ensemble	K-mers

[1] Refer to (Table. 2).
[2] Kmer, CKSNAP, PseKNC, PseDNC, SCPseKNC, EIIP, DPCP, TPCP
[3] Composition-based, position specific-based, physiochemical properties
[4] F-score ranking and sequential feature addition
[5] PRIM, RPRIM, AAPIV, RAAPIV, FV

Table 4. Performance of origin predictors on the *iORI-Epi* benchmarks. Results are reported as AUC values for three human cell lines: K562, MCF7, and HCT116.

**Predictor**	**K562**	**MCF7**	**HCT116**
iORI-Epi	0.953	0.932	0.952
Ori-FinderH	**0.961**	**0.963**	**0.987**

Table 5. Performance of origin predictors on the *iORI-EUK* benchmarks. Results are shown for multiple cell lines and benchmarks from *Homo sapiens* (*H.s*; K562, MCF7), *Mus musculus* (*M.m*; ES, MEF, P19), *Drosophila melanogaster* (*D.m*; KC, Bg3, S2), and *Arabidopsis thaliana* (*A.t*; 10-fold cross-validation and independent test set). Values are reported as (AUC, MCC) tuples. Bold indicates the highest score per column.

**Predictor**	***H.s - K562***	***H.s - MCF7***	***M.m - ES***	***M.m - MEF***	***M.m - P19***
iORI-EUK	(0.938, 0.705)	(0.882, 0.497)	(0.959, 0.764)	(0.92, 0.642)	(0.923, 0.651)
Stack-ORI	(0.935, 0.756)	(0.914, 0.661)	(0.955, 0.776)	(0.934, 0.715)	(0.939, 0.732)
ORI-Deep	(0.942, 0.762)	(0.919, 0.664)	(0.974, 0.792)	(0.943, 0.722)	(**0.992**, **0.783**)
ORI-Explorer	(0.95, 0.77)	(0.919, 0.675)	(0.962, 0.794)	(0.942, 0.721)	(0.941, 0.752)
PLANNER	(**0.957**, **0.786**)	(**0.927**, **0.696**)	(**0.976**, **0.84**)	(**0.992**, **0.783**)	(0.958, 0.781)

**Predictor**	***D.m - KC***	***D.m - Bg3***	***D.m - S2***	***A.t - 10 fold***	***A.t - Independent***
iORI-EUK	(0.945, 0.742)	(0.923, 0.656)	(0.906, 0.619)	(**0.983**, 0.876)	(0.949, 0.762)
iORI-ENST	–	–	–	(–, **0.901**)	(–, 0.824)
Stack-ORI	(0.944, 0.75)	(0.92, 0.677)	(0.905, 0.642)	–	(0.976, 0.86)
ORI-Deep	(0.936, **0.825**)	(0.938, 0.691)	(**0.977**, 0.693)	–	(**0.988**, **0.903**)
ORI-Explorer	(**0.951**, 0.771)	(0.929, 0.699)	(0.907, 0.664)	–	(0.98, 0.871)
PLANNER	(0.936, 0.743)	(**0.946**, **0.746**)	(0.935, **0.726**)	–	(0.981, 0.869)

* Additional benchmarks not included in the table columns:
* Homo sapiens Hela cell from iORI-EUK was only used by iORI-EUK with (AUC, MCC)=(0.875, 0.6).
* Pichia pastoris from iORI-EUK was only used by iORI-EUK with MCC=0.798.
* Schizosaccharomyces pombe from iORI-EUK was only used by iORI-EUK with MCC=0.997.
* Kluyveromyces lactis from iORI-EUK was only used by iORI-EUK with MCC=0.876.

Table 6. Performance of origin predictors on the *O* benchmark datasets. Results are shown for prokaryotic and yeast organisms, including *Saccharomyces cerevisiae* (*S.c*), *Schizosaccharomyces pombe* (*S.p*), *Kluyveromyces lactis* (*K.l*), and *Pichia pastoris* (*P.p*). Values are reported as (AUC, MCC) tuples. Results for O1, O3, and the Ori-Finder 3 benchmark were only reported by a single model and are included in footnotes below.

**Predictor**	**O2**	**O5 - *S.c***	**O5 - *S.p***	**O5 - *K.l***	**O5 - *P.p***
iORI-PseKNC	(0.884, 0.674)	–	–	–	–
iORI-gPseKNC	(–, **0.961**)	–	–	–	–
iRO-3wPseKNC	–	(0.808, 0.459)	(0.986, 0.929)	(0.901, 0.703)	(0.796, 0.422)
iORI-PseKNC2.0	(**0.911**, 0.756)	(0.831, 0.565)	–	–	–
iRO-PseKGCC	–	(0.812, 0.529)	–	–	(0.8, 0.484)
SefOri	–	(**0.99**, **0.98**)	(0.99, 0.94)	(**0.99**, **0.915**)	(0.89, 0.63)
XGB	(–, 0.793)	–	–	–	–
yORIpred	–	(0.917, 0.755)	(**0.994**, **0.97**)	(0.955, 0.865)	(0.946, 0.79)
iORI-ENST	(–, 0.825)	–	–	–	–
Ori-Finder 3	(0.772, 0.385)	(0.671, 0.231)	–	–	–
CNN-Based	(0.876, 0.689)	(0.935, 0.767)	–	–	–
Word Embeddings	–	(0.975, 0.94)	(0.8, 0.53)	(0.888, 0.771)	(**0.981**, **0.934**)

* O1 was only used by the BC Method with AUC=0.848.
* O3 was only used by iOri-Human with (AUC, MCC)=(0.835, 0.501).
* Ori-Finder 3 benchmark was only used by Ori-Finder 3 with (AUC, MCC)=(0.864, 0.607).

References

1. Conrad A. Nieduszynski, Shin-ichiro Hiraga, Prashanth Ak, Craig J. Benham, and Anne D. Donaldson. OriDB: a DNA replication origin database. *Nucleic Acids Research*, 35:D40–D46, 2007.
2. Yu-Hao Zeng, Zhen-Ning Yin, Hao Luo, and Feng Gao. DeOri 10.0: An updated database of experimentally identified eukaryotic replication origins. *Genomics Proteomics & Bioinformatics*, 22(5):qzae076, 2024.
3. Mei-Jing Dong, Hao Luo, and Feng Gao. DoriC 12.0: an updated database of replication origins in both complete and draft prokaryotic genomes. *Nucleic Acids Research*, 51(D1):D117–D120, 2023.
4. Theodor Sperlea, Lea Muth, Roman Martin, et al. gammaBOriS: Identification and taxonomic classification of origins of replication in gammaproteobacteria using motif-based machine learning. *Scientific Reports*, 10:6727, 2020.
5. Wei Chen, Pengmian Feng, and Hao Lin. Prediction of replication origins by calculating DNA structural properties. *FEBS Letters*, 586(6):934–938, 2012.
6. Wen-Chao Li, En-Ze Deng, Hui Ding, Wei Chen, and Hao Lin. iori-pseknc: A predictor for identifying origin of replication with pseudo k-tuple nucleotide composition. *Chemometrics and Intelligent Laboratory Systems*, 141:100–106, 2015.
7. Chang-Jian Zhang, Hua Tang, Wen-Chao Li, Hao Lin, Wei Chen, and Kuo-Chen Chou. iori-human: identify human origin of replication by incorporating dinucleotide physicochemical properties into pseudo nucleotide composition. *Oncotarget*, 7(43):69783–69793, 2016.
8. Vinod Kumar Singh, Vipin Kumar, and Annangarachari Krishnamachari. Prediction of replication sites in *saccharomyces cerevisiae* genome using DNA segment properties: Multi-view ensemble learning (MEL) approach. *BioSystems*, 163:59–69, 2018.
9. Bin Liu, Fan Weng, De-Shuang Huang, and Kuo-Chen Chou. iro-3wpseknc: identify DNA replication origins by three-window-based PseKNC. *Bioinformatics*, 34(18):3086–3093, 2018.
10. Fu-Ying Dao, Hao Lv, Hasan Zulfiqar, Hui Yang, Wei Su, Hui Gao, Hui Ding, and Hao Lin. A computational platform to identify origins of replication sites in eukaryotes. *Briefings in Bioinformatics*, 22(2):1940–1950, 2021.
11. Fu-Ying Dao, Hao Lv, Melissa J. Fullwood, and Hao Lin. Accurate identification of DNA replication origin by fusing epigenomics and chromatin interaction information. *Research*, page 9780293, 2022.
12. Dan Wang, Fei-Liao Lai, and Feng Gao. Ori-Finder 3: a web server for genome-wide prediction of replication origins in *saccharomyces cerevisiae*. *Briefings in Bioinformatics*, 22(3):bbaa182, 2021.
13. Mahwish Shahid, Maham Ilyas, Waqar Hussain, and Yaser Daanial Khan. ORI-Deep: improving the accuracy for predicting origin of replication sites by using a blend of features and long short-term memory network. *Briefings in Bioinformatics*, 23(2):bbac001, 2022.
14. Y. Yao, S. Zhang, and Y. Liang. iori-enst: identifying origin of replication sites based on elastic net and stacking learning. *SAR and QSAR in Environmental Research*, 32(4):317–331, 2021.
